# The blue mussel *Mytilus edulis* is vulnerable to the toxic dinoflagellate *Karlodinium armiger*—Adult filtration is inhibited and several life stages killed

**DOI:** 10.1371/journal.pone.0199306

**Published:** 2018-06-18

**Authors:** Sofie Bjørnholt Binzer, Regitze Benedicte Carlstedt Lundgreen, Terje Berge, Per Juel Hansen, Bent Vismann

**Affiliations:** Marine Biological Section, Department of Biology, University of Copenhagen, Helsingør, Denmark; Helmholtz-Zentrum fur Ozeanforschung Kiel, GERMANY

## Abstract

Blooms of the toxic dinoflagellates *Karlodinium armiger* and *K*. *veneficum* are frequently observed in Alfacs Bay, Spain, causing mass mortality to wild and farmed mussels. An isolate of *K*. *armiger* from Alfacs Bay was grown in the laboratory and exposed to adults, embryos and trochophore larvae of the blue mussel, *Mytilus edulis*. Adult mussels rejected to filter *K*. *armiger* at cell concentrations >1.5·10^3^ cells ml^-1^. Exposure of adult mussels (23–33 mm shell length) to a range of *K*. *armiger* cell concentrations led to mussel mortality with LC_50_ values of 9.4·10^3^ and 6.1·10^3^ cells ml^-1^ after 24 and 48 h exposure to ~3.6·10^4^ *K*. *armiger* cells ml^-1^, respectively. *Karlodinium armiger* also affected mussel embryos and trochophore larvae and feeding by *K*. *armiger* on both embryos and larvae was observed under the microscope. Embryos exposed to low *K*. *armiger* cell concentrations suffered no measurable mortality. However, at higher *K*. *armiger* cell concentrations the mortality of the embryos increased significantly with cell concentration and reached 97% at 1.8·10^3^ *K*. *armiger* cells ml^-1^ after 29 h of exposure. Natural *K*. *armiger* blooms may not only have serious direct effects on benthic communities, but may also affect the recruitment of mussels in affected areas.

## Introduction

In Alfacs Bay, Ebro Delta, Spain, in the Mediterranean, *Karlodinium veneficum* and *K*. *armiger* (syn. *Gyrodinium corsicum*) co-occur and blooms have occurred consistently since 1994 [1–4]. These blooms may last up to 3 months with maximum cell concentrations of up to 2.0·10^4^ cells ml^-1^ and are associated with mass mortality of adult mussels in both raft cultures and natural populations [1, 2, 4]. Even higher cell abundances have been reported for *K*. *veneficum* blooms with concentrations of up to 10^5^ cells ml^-1^ [5].

*Karlodinium veneficum* produces karlotoxins (KmTx) [5–8] which have been described to possess ichthyotoxic, cytotoxic and hemolytic modes of action [9–11]. In fact, all cell membranes having a certain lipid composition seem to be targets for this potent toxin, and karlotoxins can therefore harm a wide range of organisms, including other protists, metazooplankton, fish and benthic invertebrates [11, 12](reviewed in [5]). The membrane sterols of *K*. *veneficum* do not bind the toxins, which explain their immunity to their own toxins [12]. A few other organisms have been shown to be immune to *K*. *veneficum* [13, 14]. The potency of the individual KmTx toxins and the cellular toxin content depend on the specific strain and on algal growth conditions [9, 10, 15, 16]. *Karlodinium armiger* does not produce any of the known karlotoxins, but instead produces karmitoxins, which are in family with the karlotoxins, but importantly deviates by containing the longest carbon−carbon backbone discovered for this class of compounds, as well as a primary amino group [17]. Karmitoxins have been shown to be toxic to both fish gill cells as well as to copepods (*Acartia tonsa*) [17].

*Karlodinum armiger* and *K*. *veneficum* have both been shown to be mixotrophic, combining photosynthesis and food uptake [18]. The toxins seem to be involved in prey capture in both species and the content of prey is sucked out using a feeding tube [16, 19]. *Karlodinium armiger* has been shown to ingest several types of algal prey, except for diatoms, which is not used efficiently [18, 20], while *K*. *veneficum* only has been reported to ingest cryptophytes [21, 22]. Optimal prey size for *K*. *armiger* is of its own size, but it is able to ingest smaller prey and no upper prey size limit has been identified [18]. *Karlodinium armiger* has been described also to ingest and grow on copepod faecal pellets and to immobilise, kill and feed on metazoans [19, 23]. Thus, the omnivorous nature of *K*. *armiger* suggests that multiple developmental stages of mussels (i.e., embryos and larvae) will be suitable as food for *K*. *armiger*. To what extent adult mussels can serve as food for *K*. *armiger* is unknown.

Only a few studies have studied the negative effects of *Karlodinium* spp. on benthic invertebrates. Exposure of early developmental stages of the two oyster species *Crassostrea virginica* and *Crassostrea ariekensis* to a toxic *K*. *veneficum* strain (CCMP1974) led to reduced growth rates and significant mortality [24, 25]. Exposure of juveniles (1–2 cm) of the same oyster species to a slightly toxic strain led to significantly reduced growth rates, while clearance rates were not affected. The exposure of the same oyster species to a more toxic *K*. *veneficum* strain led to highly reduced clearance rates [24]. Reduced growth rates were also shown for adult *Mytilus edulis* exposed to *K*. *veneficum* (referred to as *Gymnodinium galatheanum*) [26]. The mussel *Lasaea rubra* has been shown to reject ingestion of a toxic *K*. *veneficum* strain and several mussel species experience mortality when exposed to high *K*. *veneficum* cell concentrations (at the time referred to as *Gymnodinium veneficum* [13, 27]). Thus, toxic strains of *K*. *veneficum* seem to have serious negative effects on filtration rate and survival of a number of mussel species. In contrast, evidence of negative effects of *K*. *armiger* on filtration rates and survival of mussels from controlled laboratory experiments are completely lacking.

In the present study, we investigated the effects of *K*. *armiger* on both adult and early life stages of *M*. *edulis*. More specifically, we studied: 1) the effect of *K*. *armiger* on the clearance rate of adult mussels, 2) the dependency of *K*. *armiger* cell concentration on the mortality of adult mussels and 3) the extent to which *K*. *armiger* can feed on and cause mortality to mussel embryos and trochophore larvae.

## Materials and methods

### Organisms and experimental conditions

The dinoflagellate *K*. *armiger* (strain K-0668) and the cryptophyte *Rhodomonas salina* (Strain k-0294) were grown in f/2 medium [28] based on 30–32 PSU seawater. They were grown at a temperature of 15°C and under an irradiance of ~70 μmol photons m^-2^ s^-1^ in a 14:10 h light:dark cycle. *Karlodinium armiger* was fed *R*. *salina* on a daily basis to achieve high cell concentrations for the mussel-incubations, but was starved (no prey) for at least 48 h prior to the experiments. The microalgal cultures were adapted to the above-mentioned experimental conditions for at least 7 days prior to experiments.

Adult stages of the blue mussel *M*. *edulis* (20–40 mm shell length) were dredged by an state authorized Danish research vessel in Øresund, Denmark in the spring and fall 2012, and in the winter 2015. The mussels were transported to the laboratory and kept in tanks (10 l) with fully aerated and flowing seawater (30–32 PSU) at 10°C. Two to three times per week, the mussels were fed the cryptophyte *R*. *salina* (start concentration ~ 5·10^4^ cells ml^-1^) but were starved for at least 24 h prior to experiments. Gametes (eggs and sperms) were obtained from *M*. *edulis*, kept at 10°C and fed once per week (start concentration ~ 5·10^4^ cells ml^-1^) for four months. To produce embryos and trochophore larvae, four mussels were transferred to a container with 3 litres of warmer (15°C) filtered seawater containing *R*. *salina* at a concentration of ~10^4^ cells ml^-1^. The container was aerated and an immersed centrifugal pump kept *R*. *salina* cells in suspension. With both males and females present in the container, the mussels spawned within 2–3 h. When spawning started, one female and one male were quickly removed, washed in filtered seawater (removal of *R*. *salina* cells from the mussels) and transferred to a glass beaker containing only f/2 medium. In the beaker, the mussels continued to spawn. After 30–60 minutes, fertilized eggs settled at the bottom of the beaker and were carefully collected with a plastic pipette and transferred to a glass beaker (250 ml) containing experimental f/2 medium.

### The effect of *Karlodinium armiger* on adult *Mytilus edulis*

#### Clearance rate of *Mytilus edulis* exposed to *Karlodinium armiger*

The effect of *K*. *armiger* on the clearance rate of adult *M*. *edulis* was studied. Mussels were exposed to a low and a high ecological relevant cell concentration of *K*. *armiger* (i.e., ~1.5·10^3^ and ~9.0·10^3^ cells ml^-1^). Mussels were also exposed to equivalent biovolume concentrations of *R*. *salina* (~4.0·10^3^ and ~3.5·10^4^ cells ml^-1^). The clearance rates of the non-toxic cryptophyte *R*. *salina* were used as controls.

Three mussels (30–40 mm) in each treatment were exposed to the respective algal concentration for 6 h in 6 litres of filtered seawater and the clearance rates were continuously recorded (see below). Nutrients and minerals were added to the water in all experiments to make it similar to the medium in which the algae were grown (F/2) and the aquariums received an irradiance of ~70 μmol photons m^-2^ s^-1^. Control experiments with *R*. *salina* (1.2·10^4^ cells ml^-1^) and *K*. *armiger* (1.8·10^3^ cells ml^-1^) under the same experimental conditions as above were made without mussels. This was done to measure the integrated effect of potential algal growth and sedimentation during the 6 h incubation period. The control experiments were used for correction of the clearance rates.

During the 6 h incubation, the clearance rate of *M*. *edulis* was measured using a fluorometer-controlled automatic setup, which made it possible to measure clearance rates at a constant algal concentration. The principle of measurement was published by Winter [29], improved by Riisgård and Møhlenberg [30] and Pleissner et al. [31]. The setup monitored and controlled the algal concentration in the experimental aquarium continuously. The mode of operation is based on automatic process control, which is divided into three sections: a monitoring section, a calculation section, and a regulation section. In the monitoring section water from the aquarium was continuously pumped (Masterflex model 7524–45) through a flow-through fluorometer (WetStar, Wet Labs, Oregon, USA) and returned to the aquarium, allowing continuous measurements of the fluorescence. The analogous output signal from the fluorometer was via an interface (PDM-1208LS, Measurement computing) connected to a computer equipped with data acquisition software (Labtech Notebook, ver. 12). In the calculation section, the software monitored the fluorescence (0.5 Hz) and converted it to algal concentration (cells ml^-1^) using standard curves made for both algal species (S1 Fig). In the regulation section, the algal cell concentration in the aquarium was kept constant at the pre-set algal concentration. When the algal concentration was below the pre-set concentration the acquisition software activated a pump (Gilson, Minipuls3), which pumped algae from an algal stock culture into the system until the concentration reached the pre-set concentration. The water in the aquarium was mixed by an immersed centrifugal pump (New Jet, model 400) to ensure algal concentration was homogenous throughout the aquarium, preventing the build-up of algal concentration due to the closed loop feedback acting on non-uniformly distributed algal cells. The cumulated time the algal culture pump had been active was continuously monitored by the computer and stored together with the algal concentration in the aquarium every 2.5 minutes for post experimental data analysis (for further details see [32]). Knowing the time period the algal pump had been active, the flow rate of the algal pump, the concentration of the algal culture and the algal concentration in the aquarium allowed the clearance rate (CR_obs_) to be calculated according to Eq 1[32]:

$${CR}_{obs}=\frac{1}{n∙t}\left(\frac{V_{p}∙t_{p}∙C_{s}}{C_{a}}-V_{p}∙t_{p}\right)$$ Eq 1

Where CR_obs_ = clearance rate (l h^-1^ individual^-1^), n = number of mussels, t = time (h), V_p_ = the pump rate (l h^-1^), T_p_ = time the pump had been active (h), C_s_ = concentration of the algal stock solution (cells l^-1^), and C_a_ = algal concentration in the aquarium.

The shell length of the mussels (L) in each experiment was measured and the mean value was used to estimate the dry weight of soft parts according to Eq 2 [33]:

$$dw=3.3∙10^{-6}L^{3.16}$$ Eq 2

Where dw = dry weight of soft parts (g) and L = shell length (mm).

The clearance rates were subsequently expressed as weight specific clearance rates (l h^-1^ g^-1^) according to Eq 3:

$${CR}_{ws}={\left(\frac{1}{dw}\right)}^{0.66}∙{CR}_{obs}$$ Eq 3

Where CR_ws_ = weight specific clearance rate (l h^-1^ g^-1^), dw = dry weight of soft parts (g), 0.66 = scaling exponent [34] and CR_obs_ = observed clearance rate (l h^-1^).

#### Mortality of adult *Mytilus edulis* exposed to *Karlodinium armiger*

A dose-response experiment was conducted studying the mortality of adult *M*. *edulis* exposed to six different concentrations of *K*. *armiger*, ranging from ~1·10^3^−3.6·10^4^ cells ml^-1^. The *K*. *armiger* concentrations were chosen according to a pilot study to ensure sufficient mortality among the experimental mussels. The experiment was conducted in triplicate in aquariums containing 6 l of cell suspension or, for the control, filtered seawater containing no *K*. *armiger* cells (21 aquariums in total). Filtered seawater (salinity 30 PSU), with added nutrients and minerals in concentrations corresponding to F/2 media was used for dilutions of the algal culture. The aquariums containing the different algal concentrations were placed randomly among each other. Seven mussels (shell length 23–33 mm) were added to each aquarium and their survival and behaviour were monitored after 24 and 48 h. Mussels were declared dead if shell closure failed after the mussel had been poked in the soft tissue with a needle or shells had been forced open. Mussels were declared alive if a weak shell contraction occurred or if shells were kept tightly closed when attempted forced open.

Measurement of algal concentrations, oxygen saturation, pH and temperature were conducted before mussels were added and after 24 and 48 h (S1 Table). To determine the algal concentrations in each aquarium the water were carefully mixed before 3 ml water samples were taken, fixed in acidic Lugol’s (2% final concentration) and immediately counted using a Sedgewick Rafter chamber, a minimum of 300 cells were counted. Oxygen saturation and temperature was measured using a WTW Oxi 3210 Oximeter equipped with a DurOx 325 oxygen electrode and was 91.1 ± 1.5% and 12.8 ± 0.4°C in the experiments. pH was measured with a WTW pH 3210 pH meter equipped a Sentix® 41 electrode and was 7.90 ± 0.23 in the experiments. Light of ~50 μmol photons m^-2^ s^-1^ was applied with a light:dark cycle of 14:10 h.

### The effect of *Karlodinium armiger* on *Mytilus edulis* embryos and trochophore larvae

#### Visual observations

Visual observations were made on *M*. *edulis* embryos and trochophore larvae exposed to high cell concentrations of *K*. *armiger*. Embryos and trochophore larvae were sampled post fertilisation (0–4 and 22–26 h, respectively) and added to a 24-microwell plate at a high concentration of *K*. *armiger* (4.3·10^4^ cells ml^-1^) to ensure a quick effect to be observed under the microscope. Photographs and videos of the embryos and trochophore larvae were taken using a Canon Mark III camera and an inverted microscope (Olympus CK40).

#### Predation on embryos

Embryos were exposed to different ecological relevant concentrations of *K*. *armiger* and the number of embryos was determined initially, after 2, 4 and 18 h and at the end of the experiment (29 h). The experiments were conducted in 22 ml glass vials mounted on a plankton wheel (i.e., to keep embryos and microalgae in suspension). All treatments were made in triplicates. Vials containing ~300 embryos with added *K*. *armiger* were subsequently filled to capacity with fresh medium to reach the intended concentrations of 100, 500, 1·10^3^ and 2·10^3^ cells ml^-1^. For each treatment, five sets of triplicate vials were set up for fixation at the five different incubation times. A treatment with 0 *K*. *armiger* cells ml^-1^ served as a control experiment of embryo viability. In addition, a treatment with ~1.7·10^3^ *K*. *armiger* cells ml^-1^ without embryos served as a control for algal viability. *Karlodinium armiger* is sensitive to air bubbles and the vials were therefore filled to capacity and sealed with Parafilm^®^. At each sampling (~0, 2, 4, 18 and 29 h) 3 vials from each treatment were fixed in 2% acid Lugol’s solution. At least 250 *K*. *armiger* cells were counted in a Sedgewick Rafter chamber using an inverted microscope (10x objective). At cell concentrations of ~100 cells ml^-1^ all cells in 2.5 ml were counted in a 24-microwell plate (10x objective). Embryos developed into trochophore larvae during the experiment and embryos and trochophore larvae were enumerated using an inverted microscope where all embryos and larvae were determined in 2.5 ml in a 24-microwell plate (4x objective).

### Statistics

SigmaPlot 13.0 (Systat Software) was used to check data for normality (Shapiro-Wilk) and equal variance (Brown-Forsythe) and then to perform statistical analysis. In the study of adult mussel mortality as a function of six algal concentrations the algal concentrations at start of each experiment was compared with the algal concentration at the end of experiment (48 h) using one way ANOVA followed by a post hoc Holm–Sidak test (data were normally distributed and variances were homogeneous). The mortality of *M*. *edulis* eggs and trochophore larvae at different *Karlodinum armiger* concentrations was compared to a common control experiment (i.e., no *K*. *armiger*) using one way ANOVA followed by a Dunnet post hoc test (data were normally distributed and variances were homogeneous). The effect size of differences was calculated using Cohen’s *d* and evaluated using the effect size convention given by [35]. In the dose-response experiments, the LC_50_ was obtained by fitting the mortality of adult mussels to the *K*. *armiger* concentration using sigmoid (three parameter) non-linear regressions. The two-segmented straight lines fit for critical point analysis as described by Yeager and Ultsch [36] was applied to the part of the dose-response curve < LC_50_. The critical point is determined as the intersection of the two best-fit regression lines that divide the data into two sets such that the combined residual sums of squares are minimized. In the present study, the method was used to estimate the critical point at which mortality starts to increase significantly (i.e., the first tipping point of the sigmoid curve). All results are presented as average values and are given with ± standard error (SE). In all tests the significance level was set at α = 0.05.

## Results

### The effect of *Karlodinium armiger* on adult *Mytilus edulis*

#### Clearance of adult *Mytilus edulis*

**Fig 1:**
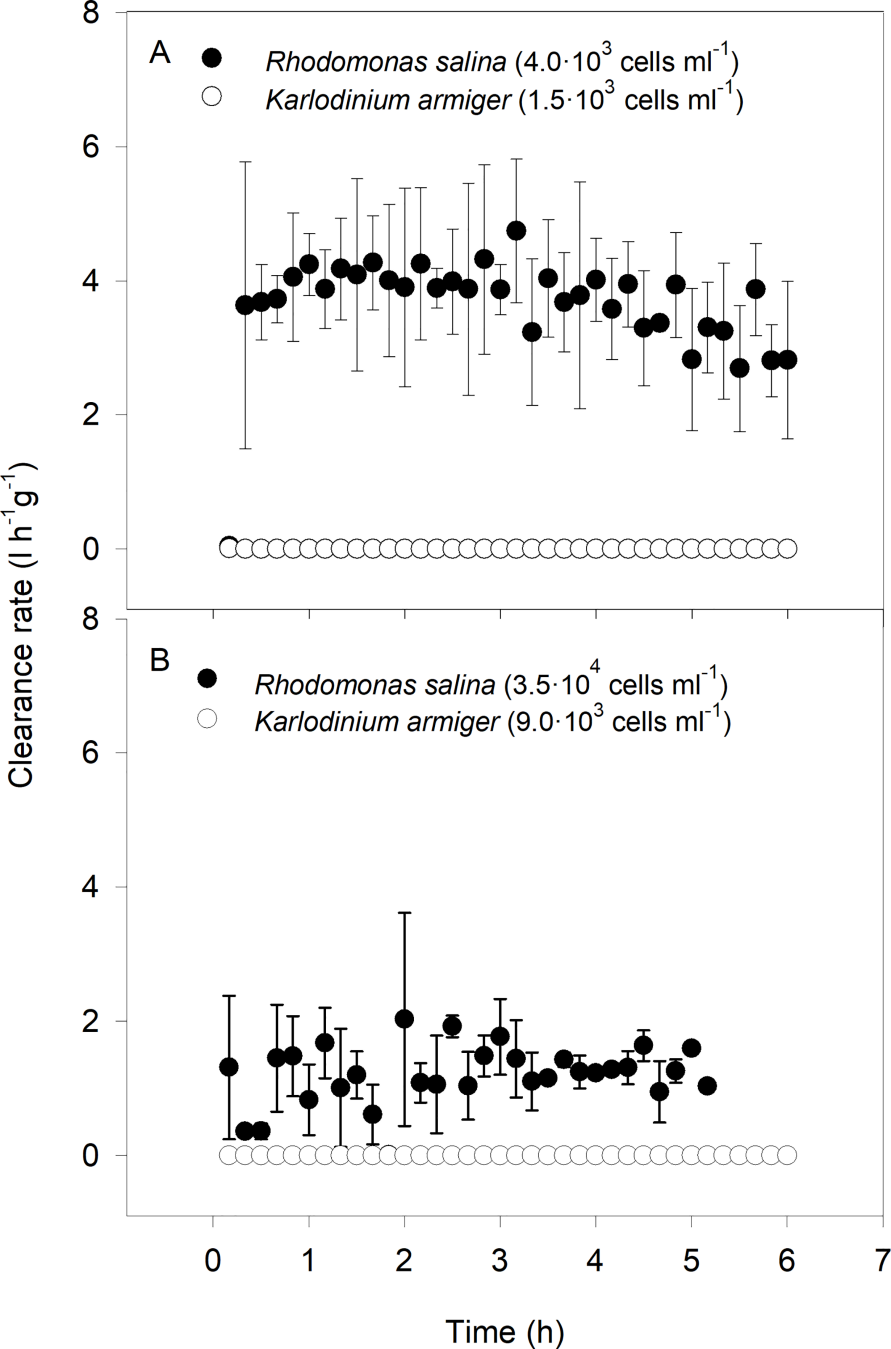
Clearance rate of *Mytilus edulis* exposed to *Karlodinium armiger* and *Rhodomonas salina*. The mussels were exposed to a low and high bio-volume equivalent concentration of each algal species. Data presented as mean values with SE bars.

*Mytilus edulis* incubated with either ~1.5·10^3^ or ~9.0·10^3^ *K*. *armiger* cells ml^-1^, failed to feed during the entire 6 h experimental period and consequently a clearance rate could not be measured (Fig 1). The mussel clearance rates of the non-toxic *Rhodomonas salina* in the treatments with ~4·10^3^ and ~3.5·10^4^ cells ml^-1^ were 3.6 ± 0.1 and 1.2 ± 0.1 l h^-1^ g^-1^, respectively (Fig 1; S2 Table). Although the mussels did not feed on *K*. *armiger*, their shells and mantle were not completely closed, while when exposed to *R*. *salina*, the mussels had their mantle and shells wide open throughout the entire experimental period and filtered the food algae. The inactive mussels in the *K*. *armiger* treatments did not excrete faeces or pseudofaeces and no mucus production was observed.

#### Mortality of adult *Mytilus edulis*

**Fig 2:**
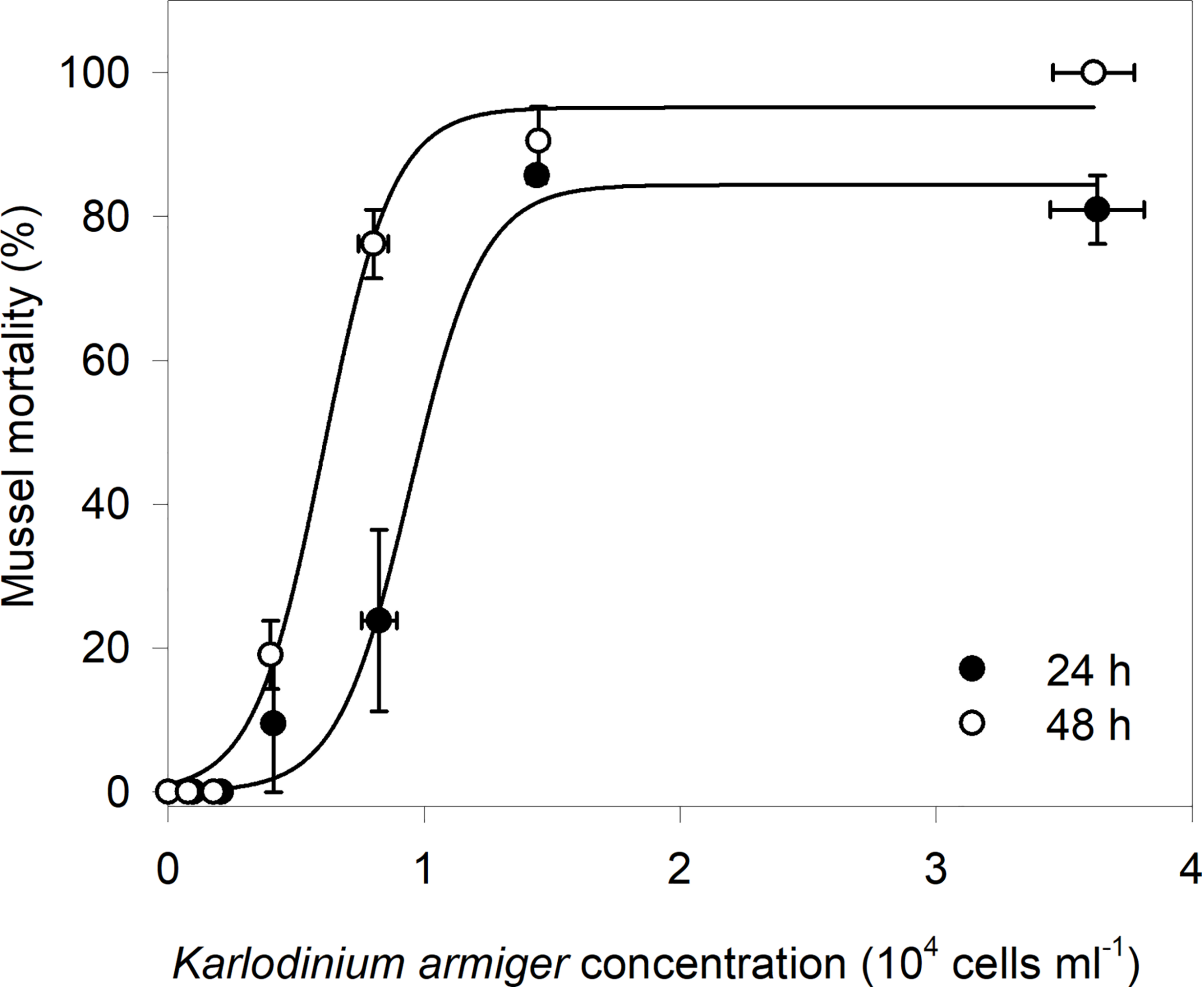
Mortality of *Mytilus edulis* exposed to six *Karlodinium armiger* concentrations for 24 and 48 h. LC_50_ values of 9.4·10^3^ ± 2.7·10^3^ cells ml^-1^ and 6.1·10^3^ ± 0.3 ·10^3^ cells ml^-1^ were found after 24 and 48 h, respectively. Data presented as mean values with SE bars.

Mussel mortality was observed after 24 h exposure to the four highest *K*. *armiger* concentrations (Fig 2; S3 Table). However, 48 h exposure was needed to observe 100% mortality when exposed to the highest *K*. *armiger* concentration (3.62·10^4^ ± 0.16·10^4^ cells ml^-1^). LC_50_ values were 9.4 ·10^3^ ± 2.7 ·10^3^ cells ml^-1^ and 6.1·10^3^ ± 0.3·10^3^ cells ml^-1^ after 24 and 48 h, respectively. The critical point analysis showed critical concentrations of 6.3·10^3^ cells ml^-1^ and 3.5·10^3^ cells ml^-1^ after 24 and 48 h respectively. Lower *K*. *armiger* concentrations resulted in reduced mortalities of the mussels after 48 h, with 91% mortality for mussels exposed to 1.45·10^4^ ± 1.59·10^4^ cells ml^-1^, 76% mortality for mussels exposed to 8.00·10^3^ ± 0.58·10^3^ cells ml^-1^ and 19% mortality to mussels exposed to 3.99·10^3^ ± 0.23·10^3^ cells ml^-1^. No mortality occured for the mussels in the control and after 24 and 48 h for the mussels exposed to the two lowest *K*. *armiger* concentrations (Fig 2, S3 Table).

During the mortality experiments, some mussels kept their shells completely closed, while others had slightly open to wide-open shells but often with the mantle retracted. The open mussels that were still alive after the exposures reacted very slowly when touched. During the 48 h incubation the *K*. *armiger* concentration did not change significantly in the four highest concentrations (p-values noted from lowest algal concentration to highest: p = 0.34; p = 0.48; p = 0.07 and p = 0.65). Hence, the mussels did not filter *K*. *armiger* cells during exposure. For the two lowest algal concentrations of 774 cells ml^-1^ and 1.76·10^3^ cells ml^-1^ we observed a significant decrease in algal concentration (p = 0.01and p = 0.02, respectively). The differences had large effect sizes (Cohens *d* = 3.47 and 2.98, respectively).

### The effect of *Karlodinium armiger* on *Mytilus edulis* embryos and trochophore larvae

#### Visual observations

**Fig 3:**
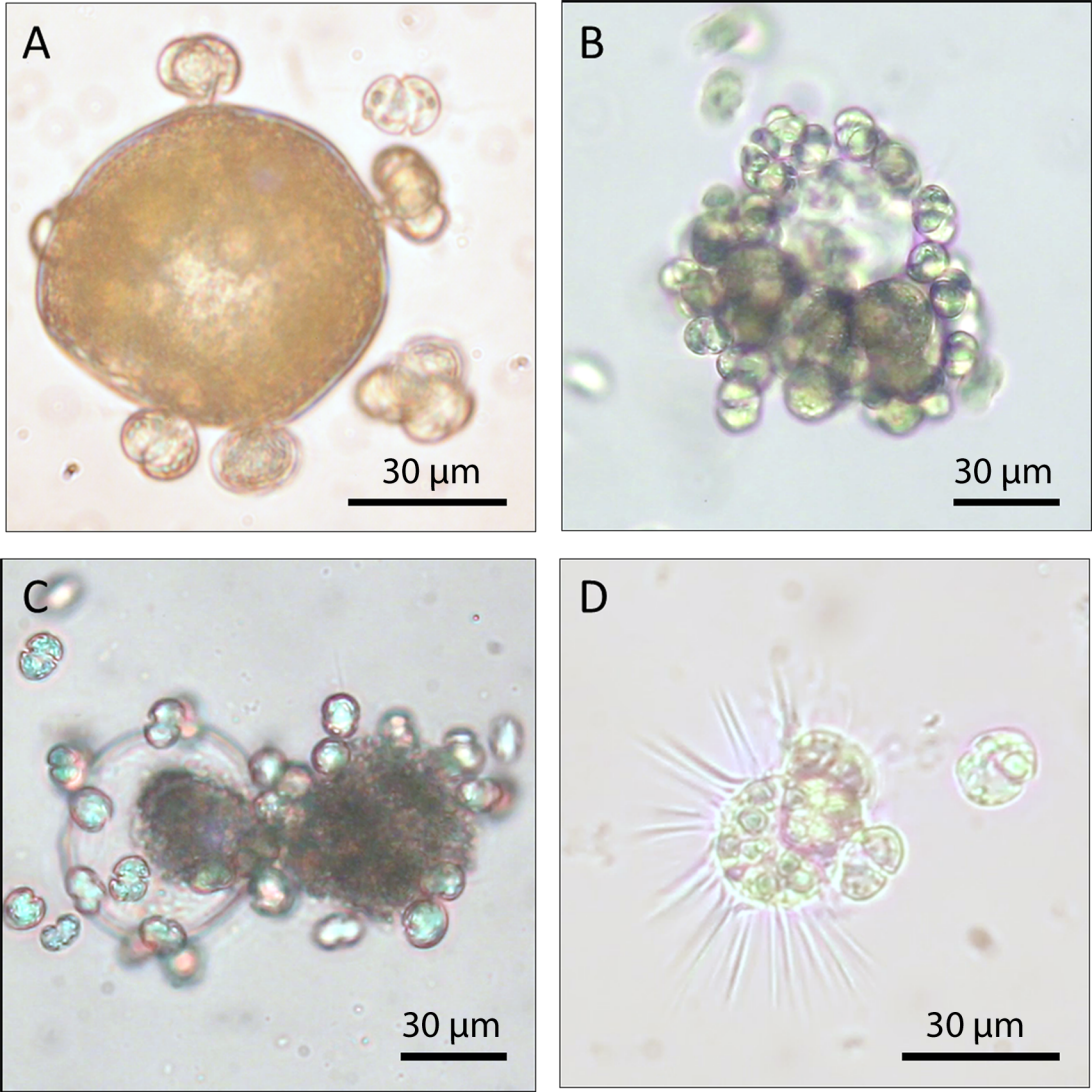
*Karlodinium armiger* tube feeding on *Mytilus edulis* embryos and trochophore larvae. Initial tube feeding by *K*. *armiger* on a mussel embryo (A). *Karlodinium armiger* cells attached to a mussel embryo causing the vitelline coat to disrupt and release of egg content (B). *Karlodinium armiger* attracted to egg content released from a disrupted embryos (C). *Karlodinium armiger* tube feeding on a mussel trochophore larva (D).

*Karlodinium armiger* was observed to feed on both mussel embryos and trochophore larvae (Fig 3A–3D, S2 and S3 Figs). Immediately after adding embryos to a culture of *K*. *armiger*, the microalgae were attracted and some of them attached to the embryos/larvae (Fig 3A). The food was observed to flow through the peduncle (feeding tube). Often, attached cells disrupted the vitelline coat resulting in the release of egg contents, leading to increased attraction by other *K*. *armiger* cells (Fig 3C, S2 Fig). The trochophore larvae seemed to be less affected by *K*. *armiger* predation than the immobile embryos. This may be due to the difficulty for *K*. *armiger* cells to attach to the trochophore larvae swimming at a high speed. Moreover, the fluid dynamics generated by the cilia, seemed to hinder attachment and forced *K*. *armiger* cells away. However, some *K*. *armiger* cells were able to attach themselves and feed on the trochophore larvae using the peduncle (Fig 3D, S3 Fig). No immobilisation effects of *K*. *armiger* seemed to occur, and the trochophore larvae kept swimming, even with several feeding *K*. *armiger* attached. We observed actively swimming trochophore larvae, where more than half of their body had been removed due to feeding from *K*. *armiger*.

#### Predation on embryos and trochophore larvae

**Fig 4:**
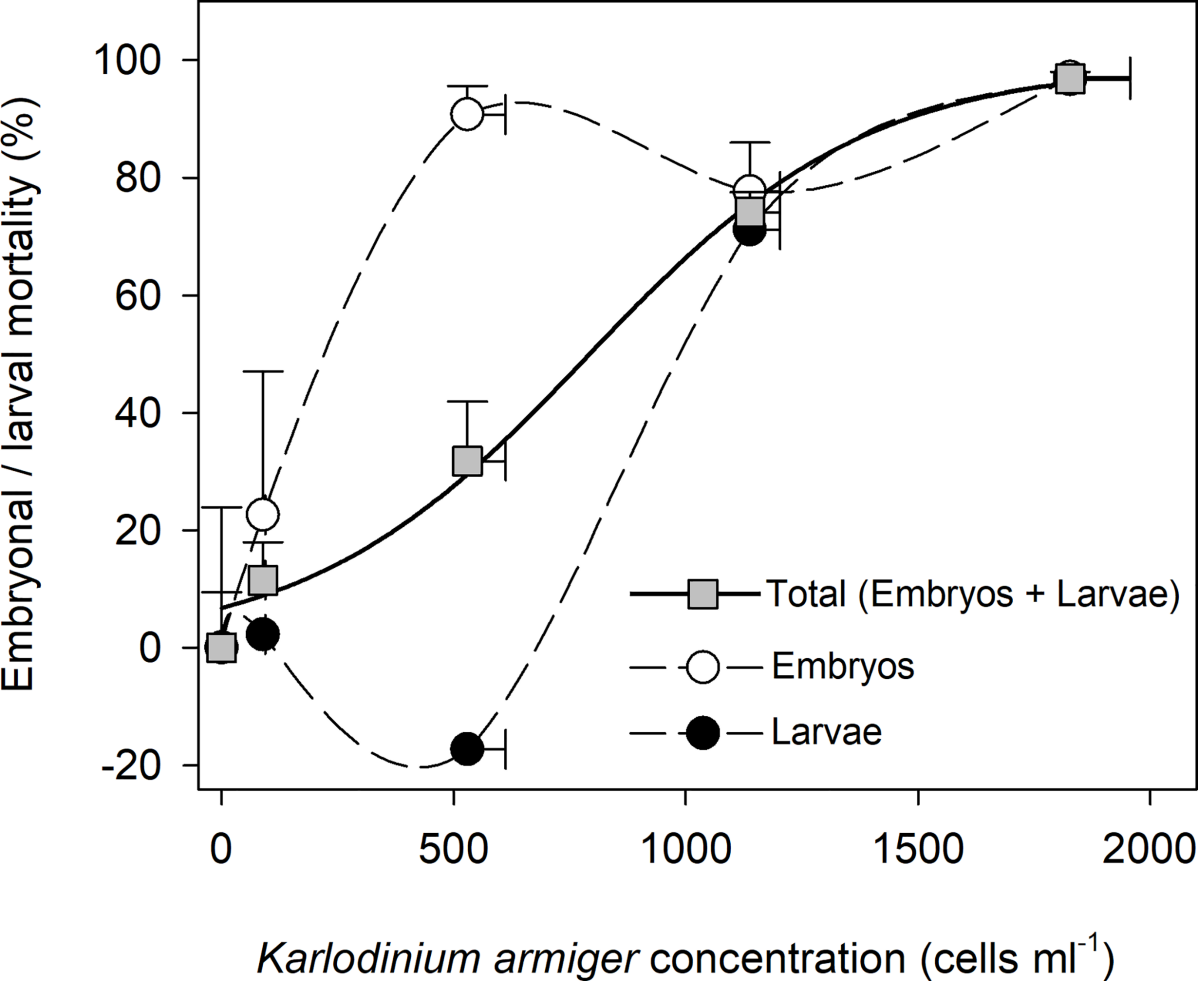
Mortality of *Mytuilus edulis* embryos and trochophore larvae after exposure to *Karlodinium armiger* at four different concentrations. Mortalities of embryos and trochophore larvae, separately and when pooled as a function of *K*. *armiger* cell concentrations. Data presented as mean values with SE bars.

The mortality of *M*. *edulis* embryos and trochophore larvae during the 29 h of incubation depended on the *K*. *armiger* cell concentration (Fig 4). During the 29 h incubation, the embryos developed into trochophore larvae and 55% of the embryos were observed to develop into trochophore larvae in the control treatment without any *K*. *armiger* cells added. If the data on embryos and trochophore larvae are pooled, exposure to 89 ± 4 cells ml^-1^ resulted in no significant mortality compared to the control (p = 0.362). At higher *K*. *armiger* cell concentrations (529 ± 82, 1.14·10^3^ ± 64 and 1.83·10^3^ ± 130 cells ml^-1^) the mortality increased with *K*. *armiger* cell concentration and was significantly different from the control (p = 0.03; p < 0.001 and p < 0.001, respectively). The effect size increased with *K*. *armiger* concentration. It was very large for the treatment with 500 cells ml^-1^ (Cohens *d* = 1.88) and huge for the two highest *K*. *armiger* concentrations (Cohens *d* = 6.04 and 8.33, respectively). A mortality of 97% was reached at 1.83·10^3^ *K*. *armiger* cells ml^-1^. Mortality data from the samples taken to time 2, 4 and 18 h are provided in S4 Table.

Mortality of embryos and trochophore larvae were monitored separately in the experiment and the data revealed that embryos were much more sensitive towards *K*. *armiger* than trochophore larvae (Fig 4). The mortality of embryos exposed to 89 *K*. *armiger* cells ml^-1^ was not significant different from the control (p = 0.72). At higher *K*. *armiger* cell concentrations (529, 1.14·10^3^ and 1.83·10^3^ cells ml^-1^) the embryo mortality (91 ± 5, 78 ± 8 and 97 ± 1%, respectively) was significantly different from the control (p = 0.008; p = 0.02 and p = 0.005, respectively) and had huge effect sizes (Cohens *d* = 3.32; 2.51 and 3.04, respectively). The mortality of trochophore larvae exposed to 89 and 529 *K*. *armiger* cells ml^-1^ was not significantly different from the control (p = 1.00 and p = 0.76). At the two highest *K*. *armiger* cell concentrations the trochophore larvae mortality (71 ± 4 and 97 ± 3%) was significantly different from the control (p = 0.01 and p = 0.001) and showed huge effect sizes (Cohens *d* = 4 and 5.53). When compared, the embryo mortality was only significantly different from the trochophore larval mortality at 529 *K*. *armiger* cells ml^-1^ (p = 0.01).

## Discussion

### Effects of *Karlodinium armiger* on adult *Mytilus edulis* clearance and mortality

*Karlodinium armiger* (strain K-0668) was not ingested by *M*. *edulis* during the 6 h exposures to cell concentrations in the range of 1.5·10^3^−9.0·10^3^ cells ml^-1^, even though the algal cell concentrations were monitored closely and determined every 2.5 min. However, we cannot rule out the possibility that a small amount of algae, below the detection limit of our method, was ingested. Previously, a few studies have investigated the effect of the closely related species *K*. *veneficum* on clearance of mussel species. The mussel *Lasaea rubra* refused to feed on a suspension of *K*. *veneficum* (strain PLY#103) during 3 h exposure, however when the alga was present in low concentrations (62 cells ml^-1^) *M*. *edulis* were able to ingest the same strain during 6 days of exposure [27, 37]. Thus, it is possible that we did not study the clearance at low enough cell concentrations and this is why we could not observe any ingestion of *K*. *armiger* cells. A significant decrease in algal concentration was observed after 48 h for the two lowest algal concentrations in the mortality experiment. We cannot rule out the possibility that the observed decrease in algal concentrations were caused by mussel ingestion since control incubations with the algae alone were not carried out. Even if so, the decreases can be calculated to correspond to an individual clearance rate of merely ~0.01 l h^-1^. However, we regard it more likely that the decrease in algal concentration was caused by lysis of *K*. *armiger* cells, which are fragile and vulnerable to disturbances (e.g., air bubbles and the air:water interface). Previously, we have observed relatively larger losses of naked dinoflagellates cells at low concentrations, simply because it takes a small amount of lysed cells to form an oily surface at the air:water interface, which when formed prevents lysis of more cells due to surface tension. Therefore, it is most likely that *K*. *armiger* at cell concentrations >800 cells ml^-1^ is not ingested by adult *M*. *edulis*. Other toxic microalgal species induce various physiological responses on *M*. *edulis*, including growth rate reduction, reduced filtration rates, shell valve closure, mucus production, reduction in byssus production, accumulation of toxins and mortality [26, 38–41]. However, it is only rarely observed that mussels completely stop feeding when exposed to a toxic algal species.

Cell concentrations of *K*. *armiger* >4·10^3^ cells ml^-1^ led to mortality of *M*. *edulis* within 24 h. A mortality as high as 100% was reached at a cell concentration of ~3.6·10^4^ cells ml^-1^ after 48 h exposure and a LC_50_ (48 h) value of 6.1·10^3^ cells ml^-1^ was observed. Algal concentrations of natural *K*. *armiger* containing blooms have been reported as high as 2.0·10^4^ cells ml^-1^ and they have been associated with mortalities of fish and shellfish [1, 2, 4]. The karmitoxins have a very sticky nature and that has probably an important influence on the concentrations needed to see an effect in controlled laboratory studies. In addition, natural blooms may last for weeks, which is much longer than the exposure times used in the present study. Taking the critical points after 24 and 48 h exposure to 3.6·10^4^ *K*. *armiger* cells ml^-1^ (6.3·10^3^ cells ml^-1^ and 3.5·10^3^ cells ml^-1^, respectively) into consideration, a long lasting natural bloom with low *K*. *armiger* concentrations is likely to cause mussel mortality. Furthermore, bloom concentrations in the same range as used here have been reported for *K*. *veneficum* blooms (10^4^–10^5^ cells ml^1^) [5], altogether suggesting that both arrested clearance and mortality of mussels are possible during natural *Karlodinium* spp. blooms. Other species of toxic algae have been observed to cause mortality in adult mussels. *Karlodinium veneficum* has been reported to kill *Lasaea rubra* and *M*. *edulis* within 3 days of exposure [13]. In addition, the mussel *Tapes semidecussatus* suffered mortality when exposed to 8·10^3^ *K*. *veneficum* cells ml^-1^, and 73% mortality was observed after 48 h exposure [2]. In the same study, another mussel species *Tapes decussatus* was exposed to the same algal strain but experienced no mortality [2].

Most often toxic algae do not cause mortalities of mussels, which is why many algal toxins, like the shellfish toxins Paralytic shellfish poisoning (PSP), Diarrhetic shellfish poisoning (DSP) and Amnesic shellfish poisoning (ASP) can accumulate in mussels (e.g., [41–43]). However, *Karlodinium* spp. are not the only toxic algae, which cause mortality to mussels. A number of harmful algae, like *Karenia* spp, *Prymnesium parvum*, and *Pfiesteria* spp, which are most often also implicated in fish kills, have been reported to kill mussels and other benthic invertebrates [44–48]. In most cases causative toxins are unknown, the exceptions being the karlotoxins, karmitoxins and prymnesins [5–7, 17, 49]. In other cases, the mortality of the mussels is suspected to be caused by algal micropredation, rather than toxins, like in the case of heterotrophic dinoflagellate *Pfiesteria shumwayae* [50]. This species has been reported to cause mortality in adults of four different mussel species ranging from <15% to > 90% mortality after 24 h exposure [45]. The mortality was found to be strain specific and to depend on whether *P*. *shumwayae* was fed algae or fish [45].

The mortality of adult *M*. *edulis* caused by *Karlodinium armiger* can potentially be obtained via several routes: *i)* internal intoxication of the mussels due to ingestion of the toxic algae, *ii*) transfer of toxins during direct contact between the two species, possible combined with micropredation by the algae on the mussel tissue or *iii*) excreted *K*. *armiger* toxins present in the water. In the present study, *M*. *edulis* was neither observed to feed on *K*. *armiger* in the clearance rate experiments nor in the mortality experiments. Therefore, the observed mortality seems highly unlikely to be caused by internal intoxication of *M*. *edulis* due to ingested *K*. *armiger* cells. Micropredation may play a role in the mortality of *M*. *edulis*. However, the immediate response from the mussels upon exposure to the *K*. *armiger* cells suggests that rather excreted/leaked toxins, or toxins released from the algal cells upon direct contact with the mussel, seem to be the most likely cause of the negative effects on adult *M*. *edulis* clearance and mortality.

### Effects of *Karlodinium armiger* on *Mytilus edulis* embryos and trochophore larvae

*Karlodinium armiger* effectively killed and ingested both embryos and trochophore larvae of *M*. *edilus* at high *K*. *armiger* cell concentrations. However, the mortalities of embryos and trochophore larvae were highly depended on the cell concentration of *K*. *armiger* and low algal concentrations resulted in no or very low mortality of both embryos and larvae. At intermediate *K*. *armiger* concentrations the embryos were highly affected, while the trochophore larvae were largely unaffected. The difference was probably related to the fact that embryos are non-motile, while the trochophore larvae swim and are difficult for the *K*. *armiger* to catch. At the high *K*. *armiger* treatments, very few larvae were observed in the experiments at any given time. This was most likely due to embryos being attacked by *K*. *armiger* and only few reached the larval stage (S4 Fig). At present it is unknown whether karmitoxins were involved or not, but it seems highly likely, since karmitoxin has been shown to impact copepods and rainbow trout gill cells [49, 51].

*Karlodinium veneficum* has not been observed to ingest mussel embryos or larvae, but toxins seem to cause mortality to oyster embryos within a day of exposure and impair larval development [25]. Exposure of oyster embryos and larvae to a toxic strain of *K*. *veneficum* (CCMp 1974) led to ~75% mortality within a day of oyster embryos being exposed to the toxic strain, whereas the exposure to a non-toxic *K*. *veneficum* strain (MD-5) did not induce any mortality [25]. Stoecker et al. [25] did not design their experiments to determine the mechanism by which *K*. *veneficum* causes mortality to oyster embryos and larvae, but judging from the existing data toxins seem to contribute to the mortality of mussels in *K*. *veneficum* as well.

### Implications

The blue mussel *M*. *edulis* represents an important suspension feeder in shallow coastal waters. Natural mussel beds and aquaculture facilities have the potential, locally to exert a strong top-down control of microalgae. The *K*. *armiger* induced arrest of *M*. *edulis* filtration reported in the present study occurred immediately and at relevant cell concentrations. Reduced mussel suspension feeding may be important for *K*. *armiger* to form blooms in nature.

Natural blooms of *K*. *armiger* will not only affect the adult mussels and persist for longer time; they will also have impact on mussel recruitment and thus may potentially have great impact on natural ecosystems. The reoccurring blooms reported from Alfacs bay, Ebro Delta, are associated with mass mortality of adult mussels in both raft cultures and natural populations [1, 52]. The spawning period of the locally cultivated mussel, *Mytilus galloprovincialis*, is from November to March [53]. This coincides in some years with the time at which *Karlodinium* spp. blooms. Alfacs Bay is the most important site for commercial mussel culturing at the Catalonian coast with an annual production of ~4·10^3^ tonnes produced by 90 mussels farms, and may be particularly vulnerable to *K*. *armiger* blooms [1, 54].

Our findings suggest that blooms of *K*. *armiger* and other *Karlodinium* spp. represent a serious threat against aquaculture and mussel production in the Mediterranean Sea and elsewhere, since *K*. *armiger* now has been reported outside the Mediterranean [55]. Nevertheless, the genus was first described in 2000 and included only three species [56]. Since then the number of species has increased to 10 [2, 56, 57, 58], a number that most likely will increase in the near future. With the development of new techniques for monitoring and species identification, it may only be a question of time before *K*. *armiger* and similar species are discovered in other locations where they can turn out to be the causative species for mortality and impaired recruitment of bivalves.

## Supporting information

S1 Fig Fluorometer calibraction curves for *Rhodomonas salina* and *Karlodinium armiger*. (PDF)

S2 Fig Video of *Mytilus edulis* embryos attacked by *Karlodinium armiger* cells. (MOV)

S3 Fig Video of *Mytilus edulis* trochophore larvae attacked by *Karlodinium armiger* cells. (MOV)

S4 Fig *Mytilus edulis* embryo and trochophore larval numbers as a function of time and *Karlodinium armiger* cell concentration. Gray squares = total (embryos + trochophore larvae), open circles = embryos and filled circles = trochophore larvae.
(PDF)

S1 Table *Karlodinium armiger* concentrations and water chemistry in the mortality of adult *Mytilus edulis* experiment. (PDF)

S2 Table Clearance rates of adult *Mytilus edulis* fed *Rhodomonas salina* or *Karlodinium armiger* at low and high algal concentrations as a function of time. Low and high algal concentrations for *R*. *salina* and *K*. *armiger* were 4.0·10^3^ and 3.5·10^4^ cells ml^-1^ and 1.5·10^3^ and 9.0·10^3^ cells ml^-1^, respectively.
(PDF)

S3 Table Mortality of adult *Mytilus edulis* after 24 and 48 h exposure to six different concentrations of *Karlodinium armiger*. (PDF)

S4 Table Mortality of *Mytilus edulis* embryos and trochophore larvae caused by *Karlodinium armiger* predation as a function of time and four different algal concentrations. (PDF)
