# Supplementary figures and images for: The blue mussel Mytilus edulis is vulnerable to the toxic dinoflagellate Karlodinium armiger—Adult filtration is inhibited and several life stages killed
Source: PLoS One. 2018 Jun 18;13(6):e0199306. doi: 10.1371/journal.pone.0199306 (PMC6005564; doi:10.1371/journal.pone.0199306)

## Fluorometer calibration curves

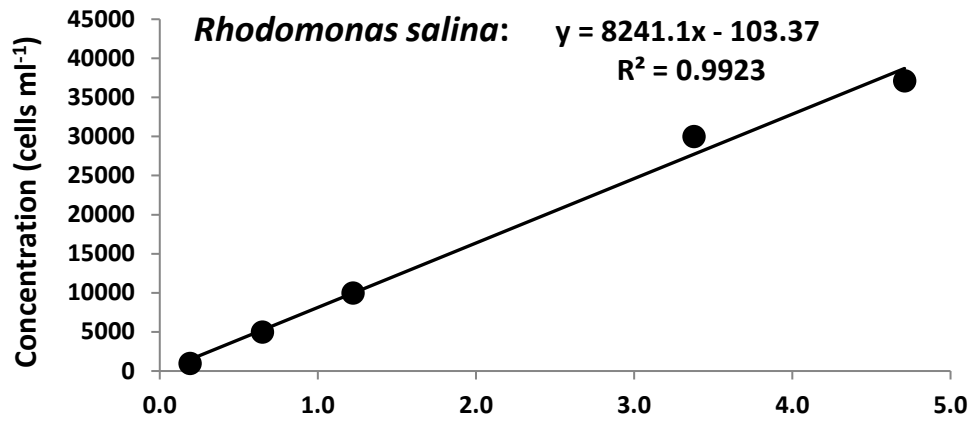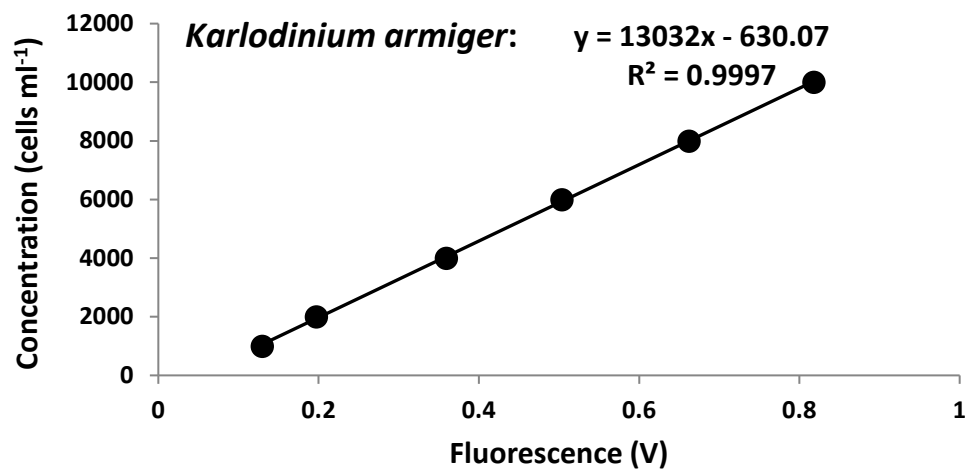

Supplement: S1 Fig — (PDF) [file pone.0199306.s001.pdf]

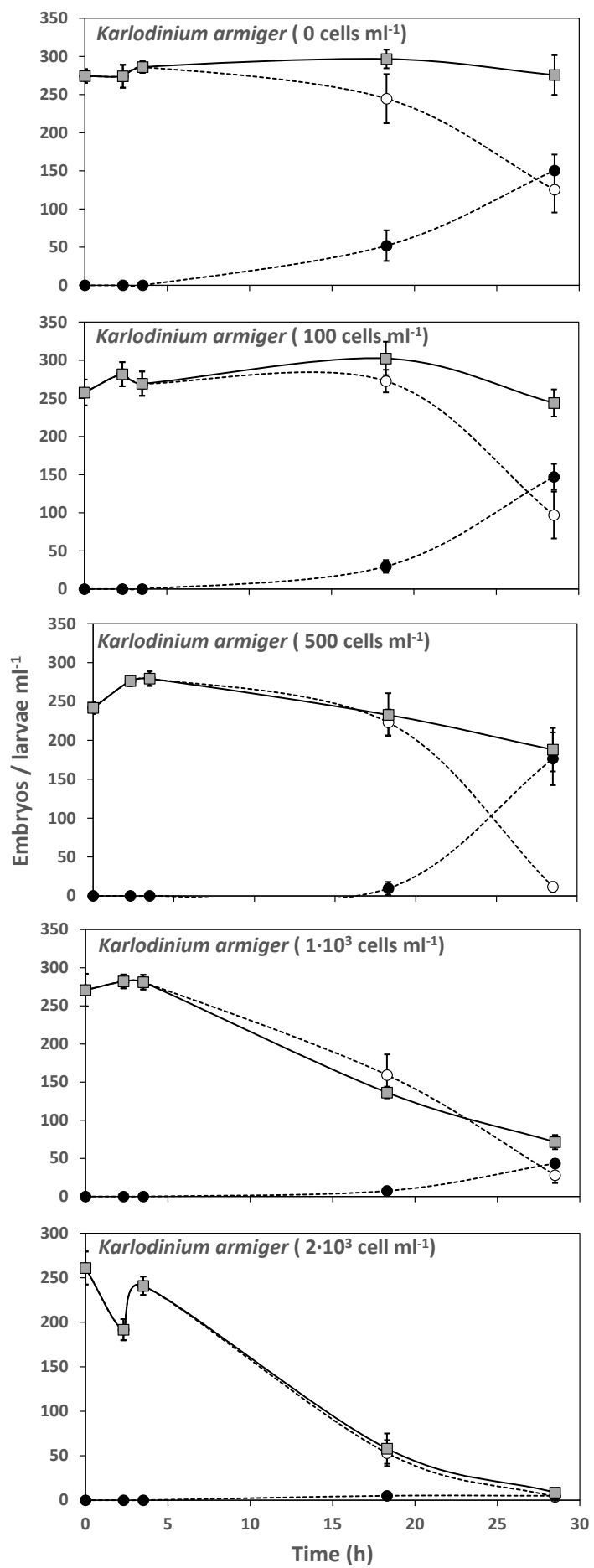

Supplement: S4 Fig — Gray squares = total (embryos + trochophore larvae), open circles = embryos and filled circles = trochophore larvae. (PDF) [file pone.0199306.s004.pdf]
