# Supplementary material for: The blue mussel Mytilus edulis is vulnerable to the toxic dinoflagellate Karlodinium armiger—Adult filtration is inhibited and several life stages killed
Source: PLoS One. 2018 Jun 18;13(6):e0199306. doi: 10.1371/journal.pone.0199306 (PMC6005564; doi:10.1371/journal.pone.0199306)
Supplement: S1 Table — (PDF) [file pone.0199306.s005.pdf]

Agal concentration and water chemistry in the mortality of adult *M. edulis* experiment					
Time (h)	Replicate	*K. armiger* (Cells ml^{-1})	pH	temperature (°C)	O_2 Saturation (%)
0	1a	0	7.795	11.9	100.1
0	1b	0	7.81	12.4	99.2
0	1c	0	7.745	14.1	95.3
0	2a	922	7.893	11.8	101.9
0	2b	1192	7.877	12.2	102.7
0	2c	814	7.889	12.3	104.1
0	3a	1978	7.913	11.9	101.3
0	3b	2350	7.924	12.4	102.9
0	3c	2019	7.903	12.3	103.7
0	4a	4213	8.048	12.1	103.3
0	4b	4538	8.031	12.7	103.5
0	4c	3510	8.027	12.2	102
0	5a	7250	8.167	12	103.4
0	5b	6691	8.15	12.5	103.8
0	5c	8000	8.161	12.3	104.2
0	6a	13960	8.281	12.5	104.8
0	6b	14222	8.272	12.5	104.3
0	6c	14129	8.274	12.7	103.5
0	7a	32417	8.518	11.7	114
0	7b	39200	8.513	13.1	113
0	7c	32273	8.515	13.6	115.2
24	1a	0	7.763	12.7	95.9
24	1b	0	7.771	13	96
24	1c	0	7.743	12.8	93.7
24	2a	969	7.776	12.9	93.9
24	2b	881	7.763	12.9	92.3
24	2c	994	7.768	12.9	93.5
24	3a	1828	7.784	12.8	91.8
24	3b	2207	7.783	13.2	91.7
24	3c	1955	7.749	13	93.4
24	4a	4475	7.843	12.9	89.5
24	4b	4275	7.84	12.9	89.4
24	4c	3620	7.845	12.7	90.9
24	5a	9675	7.936	13	88.3
24	5b	9500	7.923	12.9	87.6
24	5c	8250	7.936	12.8	89
24	6a	15600	8.034	13.2	86.6
24	6b	13875	8.025	12.8	85.5
24	6c	14542	7.987	13	81.1
24	7a	38875	8.27	12.9	82.2
24	7b	38250	8.197	12.9	76.5
24	7c	36778	8.239	12.8	81.1
48	1a	0	7.701	12.8	92.9
48	1b	0	7.708	13	93.2
48	1c	0	7.706	12.8	92.8
48	2a	450	7.666	12.9	86.4
48	2b	250	7.624	12.9	85
48	2c	498	7.644	12.9	88
48	3a	1059	7.652	12.9	84.6
48	3b	1620	7.622	13.2	80.7
48	3c	855	7.655	13	85.6
48	4a	3825	7.655	12.9	77.9
48	4b	3570	7.664	13	81.2
48	4c	3838	7.668	12.7	81
48	5a	7483	7.706	13.1	78
48	5b	7800	7.684	12.9	77.1
48	5c	7380	7.697	12.8	77.5
48	6a	14667	7.75	13.7	75.2
48	6b	14913	7.766	12.8	75.6
48	6c	14261	7.705	13	71.8
48	7a	35100	7.938	12.9	71.2
48	7b	38444	7.834	12.9	66
48	7c	34200	7.84	12.8	62.7
