# Supplementary material for: The blue mussel Mytilus edulis is vulnerable to the toxic dinoflagellate Karlodinium armiger—Adult filtration is inhibited and several life stages killed
Source: PLoS One. 2018 Jun 18;13(6):e0199306. doi: 10.1371/journal.pone.0199306 (PMC6005564; doi:10.1371/journal.pone.0199306)
Supplement: S2 Table — Low and high algal concentrations for R. salina and K. armiger were 4.0·103 and 3.5·104 cells ml-1 and 1.5·103 and 9.0·103 cells ml-1, respectively. (PDF) [file pone.0199306.s006.pdf]

Clearance rate (CR) of adult *Mytilus edulis*				
	CR at low algal concentration		CR at high algal concentration	
Time (h)	*R. salina* ($l\ h^{-1}\ g^{-1} \pm SE$)	*K. armiger* ($l\ h^{-1}\ g^{-1} \pm SE$)	*R. salina* ($l\ h^{-1}\ g^{-1} \pm SE$)	*K. armiger* ($l\ h^{-1}\ g^{-1} \pm SE$)
0.17	0.04 ± 0.03	0.01 ± 0.00	1.31 ± 1.07	0.00 ± 0.00
0.33	3.64 ± 2.14	0.00 ± 0.00	0.36 ± 0.06	0.00 ± 0.00
0.50	3.68 ± 0.56	0.00 ± 0.00	0.36 ± 0.12	0.00 ± 0.00
0.67	3.73 ± 0.35	0.00 ± 0.00	1.45 ± 0.80	0.00 ± 0.00
0.83	4.06 ± 0.96	0.00 ± 0.00	1.48 ± 0.60	0.00 ± 0.00
1.00	4.24 ± 0.46	0.00 ± 0.00	0.83 ± 0.53	0.00 ± 0.00
1.17	3.88 ± 0.59	0.00 ± 0.00	1.68 ± 0.53	0.00 ± 0.00
1.33	4.18 ± 0.76	0.00 ± 0.00	1.01 ± 0.88	0.00 ± 0.00
1.50	4.09 ± 1.44	0.00 ± 0.00	1.20 ± 0.35	0.00 ± 0.00
1.67	4.27 ± 0.70	0.00 ± 0.00	0.61 ± 0.44	0.00 ± 0.00
1.83	4.01 ± 1.14	0.00 ± 0.00	0.01 ± 0.00	0.00 ± 0.00
2.00	3.90 ± 1.48	0.00 ± 0.00	2.03 ± 1.59	0.00 ± 0.00
2.17	4.25 ± 1.14	0.00 ± 0.00	1.08 ± 0.30	0.00 ± 0.00
2.33	3.89 ± 0.30	0.00 ± 0.00	1.06 ± 0.73	0.00 ± 0.00
2.50	3.99 ± 0.78	0.00 ± 0.00	1.92 ± 0.16	0.00 ± 0.00
2.67	3.88 ± 1.58	0.00 ± 0.00	1.04 ± 0.51	0.00 ± 0.00
2.83	4.32 ± 1.41	0.00 ± 0.00	1.48 ± 0.31	0.00 ± 0.00
3.00	3.87 ± 0.38	0.00 ± 0.00	1.77 ± 0.57	0.00 ± 0.00
3.17	4.75 ± 1.07	0.00 ± 0.00	1.44 ± 0.58	0.00 ± 0.00
3.33	3.23 ± 1.10	0.00 ± 0.00	1.10 ± 0.43	0.00 ± 0.00
3.50	4.04 ± 0.88	0.00 ± 0.00	1.15 ± 0.09	0.00 ± 0.00
3.67	3.68 ± 0.74	0.00 ± 0.00	1.43 ± 0.11	0.00 ± 0.00
3.83	3.78 ± 1.69	0.00 ± 0.00	1.24 ± 0.25	0.00 ± 0.00
4.00	4.01 ± 0.62	0.00 ± 0.00	1.23 ± 0.05	0.00 ± 0.00
4.17	3.58 ± 0.75	0.00 ± 0.00	1.28 ± 0.02	0.00 ± 0.00
4.33	3.95 ± 0.64	0.00 ± 0.00	1.31 ± 0.25	0.00 ± 0.00
4.50	3.30 ± 0.86	0.00 ± 0.00	1.64 ± 0.23	0.00 ± 0.00
4.67	3.37 ± 0.11	0.00 ± 0.00	0.95 ± 0.46	0.00 ± 0.00
4.83	3.94 ± 0.78	0.00 ± 0.00	1.26 ± 0.18	0.00 ± 0.00
5.00	2.83 ± 1.06	0.00 ± 0.00	1.59 ± 0.05	0.00 ± 0.00
5.17	3.30 ± 0.68	0.00 ± 0.00	1.03 ±0.00	0.00 ± 0.00
5.33	3.25 ± 1.02	0.00 ± 0.00	-	0.00 ± 0.00
5.50	2.69 ± 0.94	0.00 ± 0.00	-	0.00 ± 0.00
5.67	3.87 ± 0.69	0.00 ± 0.00	-	0.00 ± 0.00
5.83	2.81 ± 0.54	0.00 ± 0.00	-	0.00 ± 0.00
6.00	2.82 ± 1.17	0.00 ± 0.00	-	0.00 ± 0.00
