# Supplementary material for: The blue mussel Mytilus edulis is vulnerable to the toxic dinoflagellate Karlodinium armiger—Adult filtration is inhibited and several life stages killed
Source: PLoS One. 2018 Jun 18;13(6):e0199306. doi: 10.1371/journal.pone.0199306 (PMC6005564; doi:10.1371/journal.pone.0199306)
Supplement: S3 Table — (PDF) [file pone.0199306.s007.pdf]

Mortality of adult *Mytilus edulis*			
**24 h exposure**		**48 h exposure**	
K. armiger (cells ml^{-1} ± SE)	Mussel mortality (% ± SE)	*K. armiger* (cells ml^{-1} ± SE)	Mussel mortality (% ± SE)
0.0 ± 0.0	0.0 ± 0.0	0.0 ± 0.0	0.0 ± 0.0
962 ± 75	0.0 ± 0.0	774 ± 177	0.0 ± 0.0
$2.06 \pm 0.11 \cdot 10^3$	0.0 ± 0.0	$1.76 \pm 0.29 \cdot 10^3$	0.0 ± 0.0
$4.11 \pm 0.25 \cdot 10^3$	9.5 ± 9.5	$3.99 \pm 0.23 \cdot 10^3$	19.0 ± 4.8
$8.23 \pm 0.69 \cdot 10^3$	23.8 ± 12.6	$8.00 \pm 0.58 \cdot 10^3$	76.2 ± 4.8
$1.44 \pm 0.37 \cdot 10^4$	85.7 ± 0.0	$1.45 \pm 0.31 \cdot 10^4$	90.5 ± 4.8
$3.63 \pm 1.83 \cdot 10^4$	81.0 ± 4.8	$3.62 \pm 1.59 \cdot 10^4$	100.0 ± 0.0
