# Supplementary material for: The blue mussel Mytilus edulis is vulnerable to the toxic dinoflagellate Karlodinium armiger—Adult filtration is inhibited and several life stages killed
Source: PLoS One. 2018 Jun 18;13(6):e0199306. doi: 10.1371/journal.pone.0199306 (PMC6005564; doi:10.1371/journal.pone.0199306)
Supplement: S4 Table — (PDF) [file pone.0199306.s008.pdf]

	Predation on embryos and trochophore larvae				
K. armiger (cells ml^{-1})	Embryo/larvae mortality at 0 h (% ± SE)	Embryo/larvae mortality at 2.3 h (% ± SE)	Embryo/larvae mortality at 3.5 h (% ± SE)	Embryo/larvae mortality at 18.33 h (% ± SE)	Embryo/larvae mortality at 28.5 h (% ± SE)
0.00	-5.1 ± 3.4	-5.0 ± 5.7	-9.6 ± 2.9	-13.6 ± 4.7	-5.6 ± 10.0
89 ± 4	1.3 ± 6.5	-7.9 ± 6.1	-3.2 ± 6.1	-15.8 ± 8.4	6.5 ± 6.8
529 ± 82	7.4 ± 2.9	-5.8 ± 2.4	-7.0 ± 3.5	10.9 ± 6.3	28.0 ± 10.7
$1.14 \cdot 10^3$ ± 64	-3.7 ± 8.2	-8.0 ± 3.5	-7.6 ± 3.7	47.8 ± 2.9	72.7 ± 3.6
$1.83 \cdot 10^3$ ± 130	0.0 ± 7.1	26.6 ± 4.5	7.7 ± 4.0	77.8 ± 4.7	96.7 ± 10.0
